# Are we doing enough to prevent poor-quality antimalarial medicines in the developing world?

**DOI:** 10.1186/s12889-018-5521-7

**Published:** 2018-05-15

**Authors:** Erin J. Walker, Gregory M. Peterson, James Grech, Evie Paragalli, Jackson Thomas

**Affiliations:** 1 Faculty of Health, University of Canberra, Building 12, Room D36, Kirinari Street, Bruce, Canberra, ACT 2601 Australia; 2 University of Canberra Health Research Institute (UC-HRI), Canberra, Australia; 3 Faculty of Medicine, University of Tasmania, Hobart, TAS 7001 Australia

**Keywords:** Malaria, Antimalarial, Poor-quality, Substandard, Counterfeit

## Abstract

**Background:**

Malaria is a deadly parasitic disease that affects more than 3 billion people worldwide, in predominantly resource-poor countries. Despite malaria being preventable and treatable, a large number of adults and children, mostly in Africa, die from this disease each year. One contributor to needless morbidity and mortality is the production and distribution of poor-quality antimalarial medicines; indeed, it is estimated that over 122,000 deaths of children under 5 years of age in sub-Saharan countries were caused by poor-quality antimalarial medicines, in 2013 alone.

**Discussion:**

Poor-quality medicines include those that are deliberately falsified for monetary gain and may contain incorrect amounts or even no active ingredients at all, as well as products that are inadequate due to poor compliance to conventional quality standards and medicines that have degraded over time. Across a number of studies it has been reported that 4-92% of antimalarials tested are poor quality. This represents a massive risk to the population subjected to the use of these medicines, in the form of more severe and prolonged illness, additional costs to individuals who already have very little money, and lack of confidence in treatments. The continuing circulation of poor-quality medicines results from a number of factors, including insufficient regulatory capacity in susceptible countries, inadequate funding to perform regulatory functions, poor coordination between regulatory authorities, and inefficient import/export control systems.

To combat the distribution of poor-quality medicines a number of organisations have developed guidelines for the procurement of antimalarials, and programs to educate consumers about the risks of poor-quality medicines and incentivise retailers to identify and report falsified medicines. The development of new technologies to quickly identify poor-quality medicines in the field is also essential, and some significant advances have been made.

**Conclusion:**

There has been considerable improvement in the delivery of high-quality antimalarials to those who need them; however, there is still an urgent need for a collective response by the international community, political leaders, regulatory bodies, and pharmaceutical companies. This should include political commitment for enhanced research and development funding, such as for new innovative track-and-trace field devices, and international efforts to strengthen and harmonise drug regulation practices.

## Background

Malaria, a deadly parasitic infection transmitted by mosquitoes, affects more than 3 billion people worldwide in 95 predominantly resource-poor countries [1]. In 2015, despite a reduction in incidence, 212 million new cases of malaria were reported, resulting in an estimated 429,000 deaths; the vast majority (approximately 90%) of new cases and estimated deaths were in developing countries in the World Health Organisation (WHO) African Region [2]. Over 70% of the malaria deaths in Africa (nearly 300,000 deaths) occurred in children under 5 years of age [3]. Two countries, Nigeria and Democratic Republic of the Congo, account for more than 35% of the malaria cases worldwide and a similar proportion of deaths from malaria [2]. In a 3-month period in 2016, nearby Ghana had over 2.2 million suspected malaria cases, resulting in nearly 80,000 hospital admissions, almost 50% of which were for children under 5 years of age [4].

Despite the high morbidity and mortality resulting from malaria infection, this is a preventable, curable disease. Vector control, using insecticide-treated mosquito nets and indoor residual spraying, is the main approach to prevent and reduce malaria transmission, while the use of high-quality antimalarial medicines has the potential to save hundreds of thousands of lives each year. Access to antimalarial drug therapy and the growing resistance of (i) malarial parasites to artemisinin and (ii) mosquitoes to insecticides, are significant concerns in malaria control and elimination.

An added challenge is the circulation of poor-quality antimalarials (PQAs) in the markets of endemic countries. In a sample of 39 sub-Saharan countries, it was estimated that 122,350 deaths in children under 5 years of age (representing 3.75% of all deaths for that age group) were caused by PQAs in 2013 alone [5]. This represents approximately 20% of all malaria deaths in children under 5; these were unnecessary and avoidable deaths.

### What are poor-quality medicines?

Poor-quality medicines (PQMs) can be classified as being in one of three categories: falsified, substandard or degraded. Falsified medicines are “deliberately and fraudulently mislabelled with respect to identity and/or source”, primarily created for monetary gain at the expense of patient health [6]. These medicines may contain the incorrect type or quantity of active pharmaceutical ingredients [6]. Substandard medicines are produced by legitimate manufacturers; however, the products are not compliant with quality standards. The lack of quality control may be deliberate or inadvertent. Substandard medicines contain incorrect amounts of active pharmaceutical ingredients, or may not be sufficiently bioavailable following administration. Degraded medicines begin as high-quality medicines, but either pass their expiry date or are exposed to adverse environmental conditions during transport or storage, such as extreme temperature, relative humidity or direct exposure to sunlight. If environmental conditions are not properly controlled along the entire supply route, high-quality medicines may become degraded and unwittingly, or consciously, be distributed and used. Regardless of the cause, the use of PQMs can have devastating impacts on patient health, as well as potentially promote the development of drug resistance [7], which can significantly impact entire populations requiring these medicines.

### How big is the problem?

Poor-quality antimalarials are a major impediment to malaria control, especially in resource-poor societies. A 2012 review of data from 1999 to 2010 revealed that of the samples tested, more than one-third of antimalarials supplied and distributed in South-East Asia and sub-Saharan Africa were of poor quality, with respect to failing chemical analyses or being classified as falsified [8]. Furthermore, a 2009 study found 26, 30 and 44% of antimalarials from Uganda, Madagascar and Senegal, respectively, failed quality control tests [9]. A review of reports published from 2011 to 2017 reveals that between 4 and 92% of antimalarials examined were substandard in some fashion (Table 1); the percentage of antimalarials that were found to be poor quality is indicative of the complex situation that exists, where the presence of PQAs is dependent on a combination of factors, including the cost and accessibility of high-quality antimalarials, and the presence of adequate drug control regulations in individual countries; it is clear that some countries have extensive difficulties with PQAs while other countries are impacted to a lesser degree. These data indicate a significant proportion of antimalarials being distributed in developing countries are potentially of inadequate quality.

**Table 1:** A partial summary of PQAs reported in the literature from 2011 to 2017

Drug(s)	Issue	Number of PQM failed/tested (%)	Country	Study
Artemisinin-based drugs, halofantrine	Incorrect amount of API, API absent entirely, quinine substituted for artesunate	35/59 (59%)	Burkina Faso, Chad, Cameroon, Democratic Republic of the Congo, Ghana, Kenya, Nigeria, Rwanda, Senegal	Newton et al. 2011 [34]
Artesunate	No artesunate present	1 (case report)	Equatorial Guinea	Chaccour et al. 2012 [13]
Artemisinin-based drugs, chloroquine, primaquine	Incorrect amount of API	18/77 (23%)	Guyana	Evans et al. 2012 [35]
	Incorrect packaging	30/77 (38%)		
Artesunate and amodiaquine	Incorrect amount of API	13/16 (81%)	Ghana	Affum et al. 2013 [36]
Artemisinin-based drugs	Incorrect amount of API	13/14 (92%)	Ghana	El-Duah et al. 2012 [37]
Artemisinin-based drugs, chloroquine, quinine, primaquine, amodiaquine	Incorrect amount of API	36/301 (11%)	Papua New Guinea	Hetzel et al. 2014 [38]
Artemisinin-based drugs	Incorrect amount of API	94/124 (by HPLC) (75%) 112/125 (by SQ-TLC) (89%)	Ghana and Togo	Osei-Safo et al. 2014 [39]
Artemisinin-based drugs	Incorrect amount of API	69/1737 (4%)	Tanzania	ACT Consortium Drug Quality Project Team 2015 [40]
Artemisinin-based drugs	Incorrect amount of API	206/3024 (6%)	Nigeria	Kaur et al. 2015 [41]
	Falsified (0% API)	35/3024 (1%)		
	Degradation products of API present	38/3024 (1%)		
Artemisinin-based drugs, chloroquine, quinine, sulfadoxine/pyrimethamine	Incorrect amount of API, incorrect labelling	8/28 (28%)	Cambodia	Yong et al. 2015 [42]
	Incorrect amount of API	1/7 (14%)	Indonesia	
	Incorrect amount of API, counterfeit packaging	15/30 (50%)	Laos	
	Incorrect amount of API	1/10 (10%)	Myanmar	
	Incorrect amount of API	4/8 (50%)	Thailand	
	Incorrect amount of API	1/12 (8%)	Vietnam	
Artemisinin-based drugs	Incorrect amount of API	91/291 (31%)	Cambodia	Yeung et al. 2015 [43]
	Expired at time of purchase	21/212 (9%)		
Range of antimalarial drugs	Incorrect amount of API	9/146 (6%)	Laos	Tabernero et al. 2015 [44]
Range of antimalarial drugs	Incorrect amount of API	12/37 (32%)	Afghanistan	Lalani et al. 2015 [45]
Artemisinin-based drugs	Expired at time of purchase	23/256 (8%)	Ghana	Tivura et al. 2016 [46]
	Incorrect amount of API	90/254 (35%)		
Quinine sulfate	Incorrect amount of API	7/56^a^ (12%)	Malawi	Khuluza et al. 2017 [47]
Sulfadoxine/pyrimethamine (multiple different samples)	Both stated APIs absent but other APIs present; falsified identity; incorrect amount of API			
Artemisinin-based drugs	Incorrect amount of API	11/30 (36%)	Nigeria	Izevbekhai et al. 2017 [48]

*API* active pharmaceutical ingredient, *HPLC* high-performance liquid chromatography, *SQ-TLC* semi-quantitative thin-layer chromatography; ^a^ - pharmaceuticals tested included antibiotics

### Impact of poor-quality antimalarials in “low” and “middle” income countries

The use of PQAs can have multiple consequences, including an increased risk of developing drug-resistant strains of malaria, as the sub-therapeutic doses of medicines will be ineffective in destroying all of the parasites [10], reduced consumer confidence in a specific treatment, and potentially prolonged and more severe illness [5]. Loss of consumer confidence has damaging effects on subsequent public health programs, even when they seek to deliver high-quality, effective treatments [8]. There are also significant financial consequences for patients who purchase PQAs, as they are now out of pocket for medicines that do not help, and are then forced to pay for additional treatments. It is also possible that patients may suffer unexpected adverse effects or allergic responses to components of PQAs, such as contaminants or degradation products [10, 11]. There are reports of individuals who have died or had prolonged illnesses as a direct result of taking PQAs that did not contain a therapeutic amount of the active ingredient [10, 12–14].

### Improved understanding of the problem, regulatory control and monitoring

There are many factors that contribute to the circulation of PQAs. Accurate reports of the extent of PQAs in different regions are essential in targeting all aspects of the problem, from drug production, to supply and procurement. A study in 2014 found that 63 of 104 countries (60%) with endemic malaria had no published reports of antimalarial quality [15], revealing critical gaps in essential data and an inability to identify PQA hotspots. Furthermore, there is no global system for identifying and reporting PQAs, with those in a position to respond to the presence of PQAs not being informed quickly enough to act on the problem [16].

Many of the countries that are subject to high levels of PQAs suffer serious gaps in their regulatory capacity, including shortages of adequately trained staff, inadequate funding to perform regulatory functions, poor coordination between regulatory authorities, medicine registration guidelines and assessment methods that do not meet WHO standards, and inefficient import/export control systems [17, 18]. Together, these issues make it difficult to identify and halt the movement and use of PQAs.

Lack of international supply chain regulation is another significant issue. There are many vulnerable points, from the purchase of individual drug components, production of final formulations, and the export of drugs for packaging and sale, that can be targeted or provide an opportunity for the introduction of falsified or substandard medicines [18, 19]. Currently, there are no coordinated international processes for monitoring all aspects of medicine delivery, including monitoring transport and storage conditions at all points in the supply chain to prevent degradation of genuine antimalarial medicines.

Furthermore, if falsified/substandard medicines and the individuals/companies who produced/supplied them are identified, there is little that can be done legally to stop this trade. International laws are not harmonised to prosecute in this matter and penalties are insignificant [20]. Proposals have been made to strengthen and standardise international law with regards to falsified/substandard medicines [20, 21] but any changes will take time to implement and ideally require the backing and advocacy of multiple stakeholders, including governments, public health officials and the pharmaceutical industry.

### Preventing the distribution of PQAs

There have been significant efforts to stop the trafficking of all PQMs, in operations led by INTERPOL and involving local, national and international organisations [11]. For example, Operation Pangea targets the sale of illegal medicines online, including PQMs, and Operation Mamba targets the trafficking of falsified medicines in East Africa. Whilst these operations are invaluable and have unquestionably removed falsified medicines from the supply chain [11], their continuation is essential to facilitate widespread access to high-quality medicines for life-threatening but treatable diseases. Ideally, affordable medicines will be readily available to treat malaria; expensive medicines or those that experience high demand/low supply provide the perfect setting for selling PQAs to those who cannot afford genuine medicines [19, 22].

Programs that target all aspects of PQAs, from supply and distribution to procuring legitimate, high-quality medicines, are necessary to stop the cycle of PQA delivery to patients at risk. For example, the US President’s Malaria Initiative works with a variety of local and international partners to prevent the distribution of PQAs, including: educating consumers about the risks of buying falsified and sub-standard medicines, incentivising shopkeepers to report suspected falsified medicines, and working with regulatory authorities to improve medicine quality. The US Pharmacopeial (USP) Convention has the Regulatory Standards Assistance Program, which “provides developing countries with tools to increase their capacity to test the quality of medicines for their citizens”, including reference standards “to strengthen the reliability of quality control tests” [23].

Medicine procurement and supply chains can be infiltrated with falsified medicines, so a number of non-Government organisations have guidelines regarding the procurement of malaria medicines to strengthen supply chains and ensure falsified medicines are not distributed to patients. To support the procurement of high-quality medicines, the WHO, along with global partners, developed and manage the Prequalification of Medicines Programme (PQP), aimed at assessing the quality, safety and efficacy of medicines produced by specific manufacturers [24]. This informs government and non-government organisations of manufacturers that are complying with WHO standards for medicine production, and performs ongoing inspections and monitoring to ensure medicines remain of high quality [23, 24].

### Need for additional resources

There are currently no standardised guidelines regarding the methods used and subsequent reporting measures for medicine quality; calls for a standardised set of guidelines for detecting and reporting medicine quality have been made [25, 26], and the WHO has recently published a series of guidelines for the conduct of such field surveys [27].

The ability to quickly detect falsified or substandard antimalarials in the field is essential in preventing the trade in poor-quality treatments [28]. A variety of qualitative and semi-quantitative tests are currently used to assess whether an antimalarial drug is falsified or substandard [25]. The gold standard for testing chemical content is methods such as high-performance liquid chromatography (HPLC) or mass spectrometry (MS), which require expensive equipment, trained personnel and specialist maintenance, and are not necessarily available to the countries most affected by PQAs [25]. Thus, there has been an investment in developing new methods to quickly and accurately test the quality of medicines in the field. The US Food and Drug Administration developed the counterfeit detection device version 3 (CD-3), which uses light-emitting diodes (LEDs) to detect changes in the reflected light that indicate a product may be falsified; this method is very accurate and does not destroy the tablet blister pack [29]. The counterfeit drug indicator (CoDI) uses a laser and photoresistor to detect light intensity emitted through a tablet as a result of tablet thickness, density and the wavelength of light emitted by the tablet; this provides a unique light intensity value, which can be compared to genuine tablets [30]. These methods require relatively inexpensive equipment and are simple to use, making them ideal for field surveys and ongoing monitoring at the point of care in developing countries.

Multidimensional conceptual frameworks for action have previously been described by Pribluda et al. [31] as well as the WHO [32, 33], and a commitment to follow these frameworks will aid in the delivery of high quality antimalarials to where they are needed.

## Conclusion

There have been considerable efforts to improve the delivery of high-quality antimalarials to those who need them. However, there is still an urgent need for a collective response by the international community, political leaders, regulatory bodies, and pharmaceutical companies. This should include political commitment for enhanced research and development funding, such as for new innovative track-and-trace field devices, and international efforts to strengthen and harmonise drug regulation practices.

## Abbreviations

API: Active pharmaceutical ingredient
CD-3: Counterfeit detection device version 3
CoDI: Counterfeit drug indicator
HPLC: High-performance liquid chromatography
INTERPOL: International police
LEDs: Light-emitting diodes
MS: Mass spectrometry
PQAs: Poor-quality antimalarials
PQMs: Poor-quality medicines
PQP: Prequalification of Medicines Programme
SQ-TLC: Semi-quantitative thin-layer chromatography
USP: United States Pharmacopeial
WHO: World Health Organisation
